# Shiga (Vero)-toxin producing *Escherichia coli* isolated from the hospital foods; virulence factors, o-serogroups and antimicrobial resistance properties

**DOI:** 10.1186/s13756-016-0163-y

**Published:** 2017-01-07

**Authors:** Reza Ranjbar, Mojtaba Masoudimanesh, Farhad Safarpoor Dehkordi, Nematollah Jonaidi-Jafari, Ebrahim Rahimi

**Affiliations:** 1 Molecular Biology Research Center, Baqiyatallah University of Medical Sciences, Tehran, Iran; 2 Doctor Veterinary Medicine, College of Veterinary Medicine, Islamic Azad University, Shahrekord Branch, Shahrekord, Iran; 3 Young Researchers and Elites Club, Shahrekord Branch, Islamic Azad University, Shahrekord, Iran; 4 Health Research Center, Baqiyatallah University of Medical Sciences, Tehran, Iran; 5 Department of Food Hygiene and Public Health, College of Veterinary Medicine, Shahrekord Branch, Shahrekord, Iran

**Keywords:** Shiga-toxin producing *escherichia coli*, Virulence factors, O-serogroups, Antibiotic resistance properties, Hospital foods, Iran

## Abstract

**Background:**

According to the presence of the weak, diabetic and immunosuppressive patients in hospitals, hospital foods should have a high quality and safety. Cooking a lot of foods higher than daily requirement, storage of cooked foods in an inappropriate condition and presence of nurses and servants in distribution of food to patients are the main reasons caused contamination of hospital foods. Shiga toxigenic *Escherichia coli* is one of the common cause of food poisoning in hospitals. The present research was carried out to study the distribution of virulence factors, O-serogroups and antibiotic resistance properties in STEC strains recovered from Iranian hospital food samples.

**Methods:**

Five-hundred and eighty raw and cooked food samples were collected and immediately transferred to the laboratory. *E. coli*-positive strains were subjected to PCR and disk diffusion method.

**Results:**

Thirty-nine out of 580 (6.72%) hospital food samples were contaminated with *E. coli*. Raw (20%) and cooked meat (6%) were the most commonly contaminated samples. Raw samples had the higher prevalence of *E. coli* (*P* <0.01). Samples which were collected in the summer season had the highest prevalence of bacteria (64.10%). Significant difference was seen between the prevalence of EHEC and AEEC subtypes (*P* <0.01). The most commonly detected virulence factors in both EHEC and AEEC subtypes were stx*1* and *eae*. The most commonly detected serogroups were O26 (43.75%) and O157 (25%) and there were no positive results for O103, O145, O91, O113 and O128 serogroups. *Aac* (*3*)-*IV* (100%), *CITM* (100%) and *tetA* (62.50%) were the most commonly detected antibiotic resistance genes. STEC strains harbored the highest levels of resistance against ampicillin (93.75%), gentamycin (93.75%), tetracycline (87.50%) and ciprofloxacin (81.25%). All of the STEC strains were resistant to at least 3 antibiotics, while the prevalence of resistance against more than 12 antibiotics were 12.50%.

**Conclusions:**

High presence of O157 serogroups, EHEC strains and animal-based antibiotics in cooked foods showed insufficiency of cooking time and temperature in the kitchens of hospitals. Judicious prescription of antibiotics and attentions to the principles of food safety can reduce the risk of resistant and virulent strains of STEC in hospital foods.

## Background

Access to adequate amounts of safe and healthy foods is key to supporting life and promoting good health of people. Insecure food containing pathogenic bacteria, viruses, parasites or harmful chemical elements, causes more than two-hundred diseases and disorders – ranging from diarrhea to cancers [1, 2]. Foodborne diseases cause approximately 76 million illnesses, 325,000 hospitalizations, and 5,000 deaths in the United States each year [2–4]. Observation of basic principles of food hygiene can control and eliminate the distribution of foodborne diseases. Attention to the principles of food hygiene is more urgent especially in public places such as hospitals, restaurants, kindergartens, prisons, barracks, elderly care centers and asylums.

Using from regular, nutritious, safe and accurate food diet based on the characters of patients is one of the main aspects of treatment in hospitals. Due to the general weakness and suppression of the immune system of majority of patients in hospitals, hospitals foods should have good sanitary quality. Among all pathogenic agents causing food-borne diseases and food poisoning in hospitals, *Escherichia coli* (*E. coli*) had a had a significant importance [5–8].

*E. coli* is a gram-negative, non-sporulating, flagellated, rod-shaped and facultative anaerobic bacterium which belongs to *Enterobacteriaceae* family. Shiga (vero) toxin (*Stx*)-producing *E. coli* (STEC) is a subdivision of an important pathogenic group of this bacterium named enterohemorrhagic *E. coli* (EHEC) [9–11]. STEC strains are responsible for intensive clinical syndromes like lethal hemolytic uremic syndrome (HUS), bloody and non-bloody diarrhea, thrombotic thrombocytopenic purpura (TTP) and hemorrhagic colitis (HC) [9–11]. *E. coli* strains that cause HC and HUS in humans, express high levels of Shiga toxins (stx), cause attaching-effacing (A/E) lesions in intestinal epithelial cells, and possess a specific 60-MDa EHEC plasmid are known as EHEC [9–11]. One feature EHEC and Enteropathogenic *E. coli* (EPEC) have in common is the causation of intestinal epithelial lesions known as attaching and effacing (A/E). Attaching-effacing *E coli* (AEEC) is a designation for those *E. coli* strains known to cause A/E lesions or at least carry the genes for this trait, and therefore include organisms that fall into either the EHEC or EPEC classes [9–11].

Outbreak of food poisoning, foodborne diseases, HUS, TTP and HC are associated with certain STEC O-serogroups mainly including O157, O26, O91, O103, O121, O113, O111, O145, O45 and O128 as well as untypeable groups [9–11]. Presence of latent virulence factors including Shiga toxins (*stx1* and *stx2*), intimin (*eaeA*) and hemolysin (*hlyA*) which are responsible for adhesion, colonization and invasion of bacterial cells into the intestinal walls is another factor for outbreak of foodborne diseases and disorders [9–11].

High levels of resistance in STEC strains is another important factor which increase the pathogenicity of bacteria. Unfortunately, STEC strains recovered from food stuffs and also cases of diarrhea and food poisoning harbored the high levels of resistance against commonly used groups of antibiotics including quinolones, aminoglycosides, macrolides, cephalosporins, sulfonamides, fluoroquinolones and tetracycline [9–15]. In the other hands, STEC strains of food poisoning show a high incidence of resistance (85-100%) against commonly used antimicrobial agents [9, 10, 12–14, 16, 17]. Molecular epidemiological researches showed that presence of certain antibiotic resistance genes including the genes that encode resistance against fluoroquinolone (*qnr*), trimethoprim (*dfrA1*), cephalothin (*blaSHV*), tetracycline (*tetA* and *tetB*), ampicillin (*CITM*), gentamicin (*aac* (*3*)-*IV*), sulfonamide (*sul1*), chloramphenicol (*cat1* and *cmlA*), aminoglycosides (*aadA1*), and erythromycin (*ereA*) is the most important reason for occurrence of antibiotic resistance in STEC strains [9–14, 16, 18].

According to the uncertain role of STEC strains in hospital foods and lack of epidemiological investigations in this field in Iran, the present research was carried out to study the distribution of virulence factors, O-serogroups, antibiotic resistance genes and antibiotic resistance pattern of STEC strains isolated from various types of raw and cooked hospital food samples.

## Methods

### Samples and *E. coli* isolation

From September 2013 to September 2014, a total of 580 various types of raw and cooked hospital foods including raw meat (*n* = 60), raw chicken (*n* = 60) and raw fish (*n* = 70) and cooked meat (*n* = 100), cooked chicken (*n* = 100), cooked fish (*n* = 110), and soup (*n* = 80) were randomly collected and immediately transferred to the Food Hygiene Research Center of the Islamic Azad University of Shahrekord in cooler with ice-packs. Samples were collected from the various hospitals in Isfahan province, Iran. All food samples showed normal physical characters including odor, color and consolidation. All samples were collected through 4 different seasons of the year including summer (*n* = 120), autumn (*n* = 150), winter (*n* = 160) and spring (*n* = 150).

Totally, 10-g of crushed food samples were homogenized for 2 min in 90 ml of Peptone Water (PW, Merck, Germany). Then the samples were cultured on 5% sheep blood and MacConkey agar (Merck, Germany) and incubated for 18 to 24 h at 37 °C. Colonies with the typical color and appearance of *E. coli* were picked and streaked again on blood agar plates and re-streaked on EMB agar (Merck, Germany). All plates were further incubated for 24 h at 37 °C. The green metallic sheen colonies were considered as *E. coli*. The presumptive colonies were biochemically tested for growth on triple sugar iron agar (TSI) and lysine iron agar (LIA), oxidative/fermentative degradation of glucose, citrate utilization, urease production, indol fermentation, tryptophan degradation, glucose degradation (methyl red test) and motility.

### PCR confirmation of *E. coli* strains

The colonies were further confirmed using the *16S rRNA*-based Polymerase Chain Reaction (PCR) based on the method previously described [19]. Bacterial strains were subcultured overnight in Luria-Bertani broth (Merck, Germany) and further incubated for 48 h at 37 °C. Genomic DNA was extracted from bacterial colonies using the DNA extraction kit (Fermentas, Germany) according to manufacturer’s instruction. The 10 ml bacterial DNA extract and controls were amplified with 0.5 mM primers (Forward: 5’-AGTTTGATCCTGGCTCAG-3’ and Reverse: 5’-AGGCCCGGGAACGTATTCAC-3’) (1343 bp) [19], 200 mM of each dNTP (Fermentas, Germany), 2 mM MgCl2, 10 mM KCl PCR buffer and 1.0 U Taq polymerase (Fermentas, Germany). The DNA was amplified in a programmable thermal cycler (Eppendorf, Mastercycler® 5330, Eppendorf-Netheler-Hinz GmbH, Hamburg, Germany) PCR device using the following protocol: 94 °C for 5 min, 40 cycles of 94 °C for 1 min, 55 °C for 1 min, 72 °C for 2 min, and final 72 °C for 5 min.

### PCR amplification of virulence factors, O-serogroups and antibiotic resistance genes

Table 1 shows the list of primers as well as program and condition of each reaction used for detection of O-serogroups, virulence genes and antimicrobial resistant genes [11, 20]. Programmable DNA thermo-cycler (Eppendorf Flexrcycler^2^, Germany) was used in all PCR reactions. The PCR amplification products (15 μl) were subjected to electrophoresis in a 1.5% agarose gel in 1× TBE buffer at 80 V for 30 min, stained with SYBR Green (Fermentas, Germany). All runs included a negative DNA control consisting of PCR grade water and strains of *E. coli* O157:K88ac:H19, CAPM 5933 and *E. coli* O159:H20, CAPM 6006 were used as positive controls.

**Table 1:** The oligonucleotide primers and the PCR programs used for amplification of O-serogroups, virulence factors and antibiotic resistance genes of *Escherichia coli* isolates of hospital foods

Target gene	Primer sequence (5’–3’)	PCR product (bp)	PCR programs	PCR Volume (50 μL)
O157	F: CGGACATCCATGTGATATGG R: TTGCCTATGTACAGCTAATCC	259	1 cycle: 95 ^0C^ ------------ 3 min. 30 cycle: 95 ^0C^ ------------ 20 s 58 ^0C^ ------------ 40 s 72 ^0C^ ------------ 30 s 1 cycle: 72 ^0C^ ------------ 8 min	5 μL PCR buffer 10× 2 mM Mgcl_2_ 150 μM dNTP (Fermentas) 0.75 μM of each primers F & R 1.5 U Taq DNA polymerase (Fermentas) 3 μL DNA template
O145	F: CCATCAACAGATTTAGGAGTG R: TTTCTACCGCGAATCTATC	609		
O103	F: TTGGAGCGTTAACTGGACCT R: GCTCCCGAGCACGTATAAG	321		
O26	F: CAGAATGGTTATGCTACTGT R: CTTACATTTGTTTTCGGCATC	423		
O111	F: TAGAGAAATTATCAAGTTAGTTCC R: ATAGTTATGAACATCTTGTTTAGC	406		
O91	F: GCTGACCTTCATGATCTGTTGA R: TAATTTAACCCGTAGAATCGCTGC	291	1 cycle: 94 ^0C^ ------------ 6 min. 34 cycle: 95 ^0C^ ------------ 50 s 58 ^0C^ ------------ 70 s 72 ^0C^ ------------ 55 s 1 cycle: 72 ^0C^ ------------ 10 min	5 μL PCR buffer 10× 2 mM Mgcl_2_ 150 μM dNTP (Fermentas) 0.75 μM of each primers F & R 1.5 U Taq DNA polymerase (Fermentas) 3 μL DNA template
O128	F: GCTTTCTGCCGATATTTGGC R: CCGACGGACTGATGCCGGTGATT	289		
O121	F: TGGCTAGTGGCATTCTGATG R: TGATACTTTAGCCGCCCTTG	322		
O113	F: GGGTTAGATGGAGCGCTATTGAGA R: AGGTCACCCTCTGAATTATGGCAG	771		
O45	F: CCGGGTTTCGATTTGTGAAGGTTG R: CACAACAGCCACTACTAGGCAGAA	527		
stx1	F: AAATCGCCATTCGTTGACTACTTCT R: TGCCATTCTGGCAACTCGCGATGCA	366	1 cycle: 95 ^0C^ ------------ 3 min. 34 cycle: 94 ^0C^ ------------ 60 s 56 ^0C^ ------------ 45 s 72 ^0C^ ------------ 60 s 1 cycle: 72 ^0C^ ------------ 10 min	5 μL PCR buffer 10× 2 mM Mgcl_2_ 150 μM dNTP (Fermentas) 0.75 μM of each primers F & R 1.5 U Taq DNA polymerase (Fermentas) 3 μL DNA template
stx2	F: CGATCGTCACTCACTGGTTTCATCA R: GGATATTCTCCCCACTCTGACACC	282		
eaeA	F: TGCGGCACAACAGGCGGCGA R: CGGTCGCCGCACCAGGATTC	629		
ehly	F: CAATGCAGATGCAGATACCG R: CAGAGATGTCGTTGCAGCAG	432		
aadA1	F: TATCCAGCTAAGCGCGAACT R: ATTTGCCGACTACCTTGGTC	447	1 cycle: 94 ^0C^ ------------ 8 min. 32 cycle: 95 ^0C^ ------------ 60 s 55 ^0C^ ------------ 70 s 72 ^0C^ ------------ 2 min 1 cycle: 72 ^0C^ ------------ 8 min	5 μL PCR buffer 10× 2 mM Mgcl_2_ 150 μM dNTP (Fermentas) 0.75 μM of each primers F & R 1.5 U Taq DNA polymerase (Fermentas) 3 μL DNA template
tetA	F: GGTTCACTCGAACGACGTCA R: CTGTCCGACAAGTTGCATGA	577		
tetB	F: CCTCAGCTTCTCAACGCGTG R: GCACCTTGCTGATGACTCTT	634		
dfrA1	F: GGAGTGCCAAAGGTGAACAGC R: GAGGCGAAGTCTTGGGTAAAAAC	367		
qnr	F: GGGTATGGATATTATTGATAAAG R: CTAATCCGGCAGCACTATTTA	670		
aac (3)-IV	F: CTTCAGGATGGCAAGTTGGT R: TCATCTCGTTCTCCGCTCAT	286		
sul1	F: TTCGGCATTCTGAATCTCAC R: ATGATCTAACCCTCGGTCTC	822		
blaSHV	F: TCGCCTGTGTATTATCTCCC R: CGCAGATAAATCACCACAATG	768		
CITM	F: TGGCCAGAACTGACAGGCAAA R: TTTCTCCTGAACGTGGCTGGC	462		
cat1	F: AGTTGCTCAATGTACCTATAACC R: TTGTAATTCATTAAGCATTCTGCC	547		
cmlA	F: CCGCCACGGTGTTGTTGTTATC R: CACCTTGCCTGCCCATCATTAG	698		

### Antimicrobial susceptibility testing

Pattern of antimicrobial resistance was studied using the simple disk diffusion technique. The Mueller–Hinton agar (Merck, Germany) medium was used for this purpose. Antibiotic resistance of *E. coli* strains against 23 commonly used antibiotics was determined using the instruction of Clinical and Laboratory Standards Institute guidelines [21]. Susceptibility of *E. coli* strains were tested against tetracycline (30 u/disk), mezlocillin (30 u/disk), ampicillin (10 u/disk), cefotaxime (30 μg/disk), gentamycin (10 μg/disk), ciprofloxacin (5 μg/disk), amikacin (30 u/disk), ceftazidime (30 μg/disk), imipenem (30 u/disk), cotrimoxazole (30 μg/disk), meropenem (10 μg/disk), enrofloxacin (5 μg/disk), sulfamethoxazole (25 μg/disk), trimethoprim (5 μg/disk), levofloxacillin (5 μg/disk), cefipime (30 μg/disk), streptomycin (10 μg/disk), polymyxin B (300 U/disk), vancomycine (5 μg/disk), and chloramphenicol (30 μg/disk) antibiotic agents (Oxoid, UK). All of the inoculated plates were aerobically incubated at 37 °C for 18-24 h in an aerobic atmosphere. Results were interpreted based on the instruction provided by CLSI (2014) [21]. *E. coli* ATCC 25922 was used as quality control organisms in antimicrobial susceptibility determination.

### Statistical analysis

Statistical analysis was performed using SPSS/16.0 software for significant relationships. The incidences of serogroups, virulence factors, O-serogroups and antibiotics resistance properties of *E. coli* isolated from various types of hospital food samples were statistically analyzed. Statistical significance was regarded at a *P* value < 0.05.

## Results

**Fig. 1:**
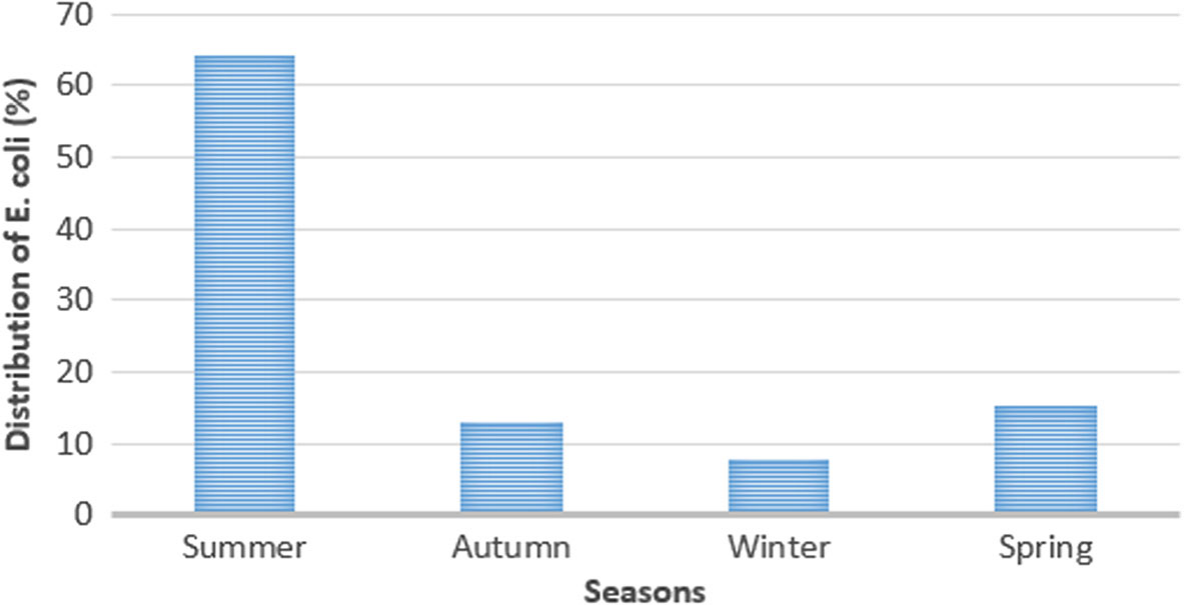
Seasonal distribution of *Escherichia coli* in the hospital food samples

Table 2 represents the total distribution of *E. coli* in various types of hospital food samples. Thirty-nine out of 580 (6.72%) hospital food samples were contaminated with *E. coli* strains. Raw meat (20%), raw chicken (16.66%) and cooked meat (6%) had the highest prevalence of *E. coli*. Statistically significant difference was seen for the prevalence of *E. coli* between raw and cooked hospital food samples (*P* <0.01). Figure 1 shows the total distribution of *E. c*oli strains in various seasons of the year based on the total numbers of 39 *E. coli* isolated. We found that hospital food samples of summer season had the highest prevalence of *E. coli* (25/39: 64.10%), while those of winter had the lowest (3/39: 7.69%). In the other hand, 25 out of 120 samples collected in summer (20.83%), 5 out of 150 samples collected in autumn (3.33%), 3 out of 160 samples collected in winter (1.87%) and 6 out of 150 samples collected in spring (4%) were positive for *E. coli*. Statistically significant difference was seen for the prevalence of *E. coli* between warm and cold season of the year (*P* <0.05).

**Table 2:** Total prevalence of *Escherichia coli* in various types of hospital food samples

Types of samples	No. samples collected	No positive strains (%)	PCR confirmation (%)
Raw meat	60	12 (20)	12 (20)
Raw chicken	60	10 (16.66)	10 (16.66)
Raw fish	70	1 (1.42)	1 (1.42)
Cooked meat	100	6 (6)	6 (6)
Cooked chicken	100	3 (3)	3 (3)
Cooked fish	110	3 (2.72)	3 (2.72)
Soup	80	4 (5)	4 (5)
Total	580	39 (6.72)	39 (6.72)

Table 3 represents the distribution of virulence factors in *E. coli* subtypes isolated from hospital food samples. Prevalence of EHEC and AEEC subtypes in the raw meat, raw chicken, cooked meat, cooked chicken, cooked fish and soup samples were 16.66% and 50%, 20% and 60%, 25% and 50%, 0% and 33.33%,0% and 50% and finally 33.33% and 66.66%, respectively. All of the EHEC strains harbored all three *stx1*, *eae* and *ehly* genes together (100%), while AEEC strains harbored variable percent of each gene. Statistically significant difference was seen between the prevalence of EHEC and AEEC subtypes (*P* <0.01). We found that from a total of 39 *E. coli* strains isolated from samples, 16 strains (41.02%) were positive for AEEC and EHEC subtypes which were considered as STEC strains. Raw fish samples had no positive strains for the EHEC and AEEC subtypes. EHEC strains which harbored all three *stx1*, *eae* and *ehly* genes together were considered as a O157 serogroup.

**Table 3:** Distribution of virulence factors in *Escherichia coli* subtypes isolated from hospital food samples

Samples (No. positive)	Subtypes	No. positive samples	Virulence genes
Raw meat (12)	Non detected	2 (33.33)	-
	EHEC	1 (16.66)	*stx1*, eae, ehly: 1 (100)
	AEEC	3 (50)	stx1: 3 (100) stx2: 1 (33.33) eaeA: 3 (100) stx1, eaeA: 1 (33.33) stx2, eaeA: 1 (33.33) stx1, stx2, eaeA: 1 (33.33)
	Total	6 (50)	
Raw chicken (10)	Non detected	1 (20)	-
	EHEC	1 (20)	stx1, eae, ehly: 1 (100)
	AEEC	3 (60)	stx1: 3 (100) stx2: 1 (33.33) eaeA: 3 (100) stx1, eaeA: 1 (33.33) stx2, eaeA: 1 (33.33) stx1, stx2, eaeA: 1 (33.33)
	Total	5 (50)	-
Raw fish (1)	Non detected	1 (100)	-
	EHEC	-	-
	AEEC	-	stx1: - stx2: - eaeA: - stx1, eaeA: - stx2, eaeA: - stx1, stx2, eaeA: -
	Total	1 (100)	-
Cooked meat (6)	Non detected	1 (25)	-
	EHEC	1 (25)	stx1, eae, ehly: 1 (100)
	AEEC	2 (50)	stx1: 2 (100) stx2: 1 (50) eaeA: 2 (100) stx1, eaeA: 1 (50) stx2, eaeA: 1 (50) stx1, stx2, eaeA: 1 (50)
	Total	4 (66.66)	-
Cooked chicken (3)	Non detected	1 (33.33)	-
	EHEC	-	-
	AEEC	1 (33.33)	stx1: 1 (100) stx2: - eaeA: 1 (100) stx1, eaeA: 1 (100) stx2, eaeA: - stx1, stx2, eaeA: -
	Total	2 (66.66)	-
Cooked fish (3)	Non detected	1 (50)	-
	EHEC	-	-
	AEEC	1 (50)	stx1: 1 (100) stx2: - eaeA: 1 (100) stx1, eaeA: 1 (100) stx2, eaeA: - stx1, stx2, eaeA: -
	Total	2 (66.66)	-
Soup (4)	Non detected	-	-
	EHEC	1 (33.33)	stx1, eae, ehly: 1 (100)
	AEEC	2 (66.66)	stx1: 2 (100) stx2: 1 (50) eaeA: 2 (100) stx1, eaeA: 1 (50) stx2, eaeA: 1 (50) stx1, stx2, eaeA: 1 (50)
	Total	3 (50)	-

Table 4 shows the total distribution of O-serogroups in the STEC strains isolated from various types of hospital food samples. We found that O26 (43.75%) and O157 (25%) were the most commonly detected serogroups in the STEC strains of hospital food samples. There were no positive results for the O103, O145, O91, O113 and O128 serogroups. Statistically significant differences were seen for the prevalence of STEC O-serogroups between various types of samples (*P* <0.05). Totally, raw chicken and soup samples harbored the most variable types of O-serogroups.

**Table 4:** Total distribution of O-serogroups in the Shiga toxigenic Escherichia coli strains isolated from various types of hospital food samples

Samples (No. STEC strains)	Distribution of O-serogroups (%)									
	O157	O26	O103	O111	O145	O45	O91	O113	O121	O128
Raw meat (4)	1 (25)	2 (50)	-	1 (25)	-	-	-	-	-	-
Raw chicken (4)	1 (25)	1 (25)	-	1 (25)	-	-	-	-	1 (25)	-
Cooked meat (3)	1 (33.33)	1 (33.33)	-	-	-	1 (33.33)	-	-	-	-
Cooked chicken (1)	-	1 (100)	-	-	-	-	-	-	-	-
Cooked fish (1)	-	1 (100)	-	-	-	-	-	-	-	-
Soup (3)	1 (33.33)	1 (33.33)	-	-	-	-	-	-	1 (33.33)	-
Total (16)	4 (25)	7 (43.75)	-	2 (12.50)	-	1 (6.25)	-	-	2 (12.50)	-

Total distribution of antibiotic resistance genes in the STEC strains isolated from various types of hospital food samples is shown in Table 5. We found that *aac* (*3*)-*IV* (100%), *CITM* (100%), *tetA* (62.50%), *dfrA1* (56.25%) and *sul1* (56.25%) were the most commonly detected antibiotic resistance genes. The less commonly detected antibiotic resistance genes were *cmlA* (12.50%), *cat1* (25%) and *tetB* (31.25%). Statistically significant differences were seen for the prevalence of antibiotic resistance genes between various types of samples (*P* <0.05).

**Table 5:** Total distribution of antibiotic resistance genes in the Shiga toxigenic Escherichia coli strains isolated from various types of hospital food samples

Samples (No. STEC strains)	Distribution of antibiotic resistance genes (%)										
	aadA1	tetA	tetB	dfrA1	qnr	aac (3)-IV	sul1	blaSHV	CITM	cat1	cmlA
Raw meat (4)	1 (25)	1 (25)	-	1 (25)	1 (25)	4 (100)	2 (50)	1 (25)	4 (100)	1 (25)	-
Raw chicken (4)	2 (50)	2 (50)	1 (25)	2 (50)	1 (25)	4 (100)	4 (100)	2 (50)	4 (100)	3 (75)	2 (50)
Cooked meat (3)	1 (33.33)	3 (100)	2 (66.66)	2 (66.66)	2 (66.66)	3 (100)	1 (33.33)	1 (33.33)	3 (100)	-	-
Cooked chicken (1)	-	1 (100)	1 (100)	1 (100)	1 (100)	1 (100)	1 (100)	-	1 (100)	-	-
Cooked fish (1)	-	1 (100)	-	1 (100)	-	1 (100)	-	-	1 (100)	-	-
Soup (3)	1 (33.33)	2 (66.66)	1 (33.33)	2 (66.66)	1 (33.33)	3 (100)	1 (33.33)	1 (33.33)	3 (100)	-	-
Total (16)	5 (31.25)	10 (62.50)	5 (31.25)	9 (56.25)	6 (37.50)	16 (100)	9 (56.25)	5 (31.25)	16 (100)	4 (25)	2 (12.50)

**Fig. 2:**
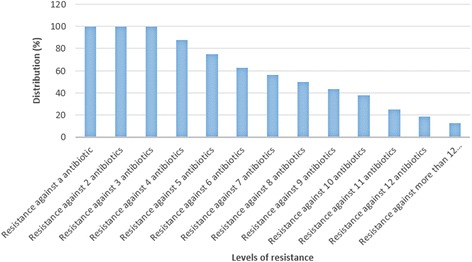
Prevalence of multi-drug resistant strains of Shiga toxigenic *Escherichia coli* isolated from hospital food samples

Table 6 indicates the antibiotic resistance pattern of the STEC strains of various types of hospital food samples. STEC strains of our investigation harbored the highest levels of resistance against ampicillin (93.75%), gentamycin (93.75%), tetracycline (87.50%), ciprofloxacin (81.25%) and amikacin (75%). Resistance of STEC strains of cooked samples against human-based antibiotics was entirely higher than animal-based antibiotics. Statistically significant differences were seen for the prevalence of antibiotic resistance between various types of samples (*P* <0.05). Figure 2 represents the prevalence of multi-drug resistance in the STEC strains of hospital food samples. We found that all of the STEC strains of our investigation were resistant to at least 3 antibiotics, while prevalence of resistance against ten, eleven, twelve and more than twelve antibiotics were 37.50%, 25%, 18.75% and 12.50%, respectively.

**Table 6:** Antibiotic resistance pattern of Shiga toxigenic *Escherichia coli* strains isolated from various types of hospital food samples

Antibiotic resistance	Samples (No. STEC strains)						
	Raw meat (4)	Raw chicken (4)	Cooked meat (3)	Cooked chicken (1)	Cooked fish (1)	Soup (3)	Total (16)
Tetracycline	4 (100)	4 (100)	2 (66.66)	1 (100)	1 (100)	2 (66.66)	14 (87.50)
Ampicillin	4 (100)	4 (100)	3 (100)	1 (100)	1 (100)	2 (66.66)	15 (93.75)
Gentamycin	4 (100)	4 (100)	2 (66.66)	1 (100)	1 (100)	3 (100)	15 (93.75)
Amikacin	2 (50)	3 (75)	2 (66.66)	1 (100)	1 (100)	3 (100)	12 (75)
Imipenem	-	-	-	1 (100)	-	1 (33.33)	2 (12.5)
Meropenem	-	-	-	-	-	1 (33.33)	1 (6.25)
Mezlocillin	-	-	1 (33.33)	1 (100)	1 (100)	1 (33.33)	4 (25)
Sulfamethoxazole	1 (25)	3 (75)	1 (33.33)	1 (100)	1 (100)	2 (66.66)	9 (56.25)
Cefotaxime	-	-	-	1 (100)	-	2 (66.66)	3 (18.75)
Ciprofloxacin	2 (50)	3 (75)	3 (100)	1 (100)	1 (100)	3 (100)	13 (81.25)
Enrofloxacin	2 (50)	4 (100)	-	-	-	1 (33.33)	7 (43.75)
Cotrimoxazole	-	-	2 (66.66)	1 (100)	1 (100)	2 (66.66)	6 (37.50)
Ceftazidime	-	-	1 (33.33)	1 (100)	1 (100)	2 (66.66)	5 (31.25)
Trimethoprim	1 (25)	2 (50)	2 (66.66)	1 (100)	1 (100)	1 (33.33)	8 (50)
Cefipime	-	-	2 (66.66)	1 (100)	1 (100)	2 (66.66)	6 (37.50)
Levofloxacillin	-	-	2 (66.66)	1 (100)	1 (100)	3 (100)	7 (43.75)
Streptomycin	1 (25)	2 (50)	-	-	-	-	3 (18.75)
Vancomycine	-	-	1 (33.33)	1 (100)	1 (100)	2 (66.66)	5 (31.25)
Polymyxin B	-	-	1 (33.33)	1 (100)	1 (100)	1 (33.33)	4 (25)
Chloramphenicol	1 (25)	2 (50)	-	-	-	-	3 (18.75)

## Discussion

As far as we know the present investigation is the first prevalence report of the isolation of *E. coli* strains from raw and cocked hospital foods as well as molecular characterization and antimicrobial resistance properties of their STEC strains in the world. We found that 6.72% of hospital food samples were contaminated with *E. coli* strains and the prevalence of STEC strains were 41.02% which was considerable high. Gram-negative bacteria like *E. c*oli are responsible for 30-70% of hospitalization in developed countries and *E. coli* was the most common etiologic gram-negative organism in the hospital environment [22, 23].

There were some probable reasons for the high prevalence of *E. coli* and also STEC strains in the hospital food samples. At first. high-volume food production and long process of catering caused several problems such as lack of adequate precision and accuracy in the preparation and washing of raw materials and even their well cooking, lack of enough time and even temperature for cooking of raw materials, cooling of foods during processing, lack of reaching of sufficient heat to the center of meat and other food materials, lack of enough time to withdraw meat from the frozen state and finally cooking of meat and its products more than the daily requirement and then their storage at improper temperature and conditions. These mentioned circumstances maybe lead to survival and even growth of pathogenic microorganisms in food stuffs. At second, using from unsanitary and also contaminated equipment and dishes for production of foods in hospitals. At third, presence of nurses and servants, which maybe the sources of dangerous pathogenic agents, in the distribution of food to patients. Prevalence of *E. coli* in raw and cooked fish samples were 1.42% and 2.72%, respectively. Presence of powerful competitor microflora in raw fish and also their destruction and addition of *E. coli* strains derived from contaminated staffs of the hospital’s kitchen are two main reasons for above findings. Because the large numbers of *E. coli* isolates recovered from raw meat, proper preparation of the raw meat can eliminate the distribution of bacteria. Unfortunately, basic principles of meat inspections were not observed in Iranian slaughterhouses. Therefore, close contact of animal carcasses with each other and even slaughterhouse floor, blood, content of the digestive tract and wool and skin of slaughtered animal caused transmission and distribution of pathogenic agents like *E. coli* to meat of slaughtered animals. Besides, the role of possible colonizers such as meat inspectors, butchers and miscellaneous people which mainly have come into the slaughterhouse for buying of meat and finally animals like rats, cats and birds which have been entered from outside the slaughterhouse as a sources of pathogenic *E. coli* should not be overlooked. Survival of STEC strains of raw food samples even after cooking procedure and occurrence of cross contamination after cooking procedure are two important routes of hospital foods contamination.

We found marked seasonality in distribution of *E. coli* strains in hospital food samples. High incidence of *E. coli* strains in summer season (64.10%) could be related to the low levels of individual hygiene in this season. The higher prevalence of STEC strains may be related to higher growth of them in hot seasons of the year. Of studies that have been conducted in this field [24–26], all have shown a seasonal distribution for *E. coli* with a higher prevalence of strains in warmer months of the year [24–26].

The results of the current study showed that resistant and virulence STEC strains had the high prevalence in the hospital food samples. Limit studies have been conducted in this field. Ifeadike et al., [27] revealed that the prevalence of *E. coli* strains in food handlers of Nigerian hospitals were 1.8% which was lower than our findings in hospital foods. They concluded that poor and faulty food-handling practices have been identified as the leading cause of the majority of food-borne disease in hospitals. Ha et al., [7] in a research which was conducted in order to study the contamination rate of raw foods in schools, factories, and hospitals of Vietnam reported that the prevalence of *E. coli* in raw poultry, meat, fish and vegetable samples were 45, 21.3, 6.6 and 18.5%, respectively which all were higher than our results.

STEC strains of our investigation harbored resistance against multiple antibiotics. The results of disk diffusion method obtained from STEC strains of our investigation were also confirmed by the presence of specific antibiotic resistance genes encode resistance against determined antibiotics. Stewardson et al., [5] reported that 92% of all food samples which were collected from hospitals in Switzerland were positive for Extended-Spectrum-Beta-lactamase-producing-Enterobacteriaceae (ESBL-PE). ESBL-producing *E. coli* was the most commonly detected (44.77%). They showed 6.45% of eligible food handlers were positive for ESBL-PE and prevalence of *E. c*oli strains among ESBL-PE strains were 83.33%. ESBL-PE strains were generally not multidrug resistant, with 100, 90, 87, 79, and 98% of strains susceptible to meropenem, gentamicin, ciprofloxacin, cotrimoxazole, and fosfomycin, respectively. Antibiotic resistance-based finding of Stewardson et al., [5] study was in contrast with our results which showed the high prevalence of resistance and also antibiotic resistance genes. This part of our study was similar with those of India [28] (high prevalence of resistance against erythromycin, cephalothin, amikacin, kanamycin and gentamicin antibiotics), South Africa [29] (high presence of *CITM*, *blaSHV* and *tetA* antibiotic resistance genes), Korea [30] (high prevalence of resistance against ampicillin, tetracycline, streptomycin and amikacin antibiotics) and Mexico [31] (high presence of *tet*, *blaSHV*, *qnr* and *aac* (*3*)-*IV* antibiotic resistance genes and also high prevalence of resistance against ampicillin, trimethoprim-sulfamethoxazole, chloramphenicol and cephalotine antibiotics). Momtaz et al., [20] reported that *aac* (*3*)-*IV* (68.03%), *sul1* (82.78%), *blaSHV* (56.55%), *aadA1* (60.65%) and *tetA* (51.63%) and also resistance against tetracycline (86.88%), penicillin (100%), gentamycin (62.29%) and streptomycin (54.91%) were the most commonly reported antibiotic resistance-based finding of STEC strains of diarrheic patients which was similar to our results.

STEC strains of hospital food samples and especially cooked samples harbored the high levels of resistance against human-based antibiotics such as meropenem, levofloxacillin, mezlocillin, cefipime, polymyxin B, cefotaxime, ciprofloxacin, cotrimoxazole, ceftazidime and imipenem which can indirectly confirm their anthropogenic origin of these strains. The prevalence of antibiotic resistance genes and especially those that were encode resistance against human-based antibiotics were also high among STEC strains recovered from cooked hospital food samples which also can indirectly approved the transmission of anthropogenic STEC strains probably from staffs of hospital kitchens to foods after cooking process. Prevalence of resistance against chloramphenicol antibiotic in the STEC strains of meat and chicken samples of our investigation were 25 and 50%, respectively. There were no positive results in other raw and also all of the cooked food samples. The Iranian Food and Drug Administration (FDA) listed chloramphenicol as a forbidden antibiotic for treatment of diseases of animals and poultries. It is because of prescription of this antibiotic may cause dangerous effects on animals and even humans who use from their edible sources like meat and milk. The presence of high chloramphenicol resistance showed its irregular and unauthorized use in veterinary treatment and especially field of poultry in Iran. Veterinary practitioners of the field of poultry use from this antibiotic as a primary choice for treatment of diseases. Therefore, in a very short period of time, antibiotic resistance appears. Similar results have been reported by Momtaz and Jamshidi [32], Ranjbar et al., [33] and Colello et al., [34]. Momtaz and Jamshidi [32] reported that 73.17% of STEC strains recovered from poultry meat samples were resistant against chloramphenicol which was higher than our findings. Ranjbar et al., [33] found that the prevalence of resistance against chloramphenicol in the STEC strains recovered from various types of food samples were 21.95% which was considerable. Colello et al. [34] showed that majority of STEC strains in the Argentina harboured the gene that encodes resistance against chloramphenicol.

The most commonly detected serogroups in the STEC strains of hospital food samples of our study were O26 and O157. Presence of O157 serogroup showed that some of our strains had animal-based origin. O157 serogroup is predominant in foods with animal origin. STEC strains of raw foods with animal origins like those of meat and chicken were remained even after cooking of foods. Presence of this serogroup. High prevalence of O157 and O26 serogroups have been reported previously from Iran (Hemmatinezhad et al., [35]), Nigeria (Mamza et al., [36]), Canada (Neher et al., [37]) and Turkey (Avaz et al., [38]). Dehkordi et al., [11] reported similar profile for distribution of STEC O-serogroups. They showed that total prevalence of O157, O145, O128, O121, O113, O111, O103, O91, O45 and O26 serogroups in the STEC strains of food stuffs were 26, 6, 8, 4, 6, 6, 6, 4, 4 and 12%, respectively, which was similar to our findings.

Another part of our investigation focused on the distribution of *stx1*, *stx2*, *eaeA* and *ehly* virulence factors in the *E. coli* strains of hospital foods. High presence of these factors in EHEC and AEEC subtypes showed their high pathogenicity for human and especially patients. Simultaneous presence of *stx1* and *eaeA* and *stx2* and *eaeA* virulence factors in some strains of *E. coli* of hospital foods indicated the important public health problem facing Iranian hospitals and health centers which can attributed to occurrence of dangerous food poisoning and STEC-based food-borne diseases in hospitals. Simultaneous presence of *stx1* and *eaeA* and *stx2* and *eaeA* virulence factors have been reported previously [39–41]. In a study which was conducted by Momtaz et al., [42] the prevalence of EHEC and AEEC subtypes in *E. coli* strains of food samples were 15.06 and 49.31%, respectively. They showed that all of the EHEC strains were positive for all stx*1*, *eaeA* and *ehly* virulence genes, while the prevalence of these genes in AEEC subtypes were 77.77, 13.88 and 55.55%, respectively. Higher prevalence of AEEC subtypes was also reported by various investigations [9–11, 20, 32, 33]. Prevalence of AEEC subtypes in the studies of Momtaz et al., [9] and Dehkordi et al., [11] were 79.41 and 62%, respectively which were higher than EHEC subtype in both studies. Momtaz et al., [10] reported that the prevalence of AEEC subtypes in the *E. coli* strains recovered from the meat samples of beef, sheep, goat and camel species were 45.54, 44.87, 39.02 and 50.00%, respectively which were higher than those of EHEC. Momtaz et al., [20] reported that the prevalence of AEEC subtypes among the *E. coli* strains of Iranian diarrheic patients had a range of 47 to 75% which was entirely high.

## Conclusions

In conclusions, we identified a large number of O-serogroups, virulence factors, antibiotic resistance genes and pattern of antibiotic resistance in STEC strains recovered from hospital foods. Raw meat, raw chicken and cooked meat, summer season, *stx1* and *eaeA* virulence factors, AEEC subtype, O26 and O157 serogroups, *aac* (*3*)-*IV*, *CITM*, *tetA*, *dfrA1*) and *sul1* antibiotic resistance genes, resistance against ampicillin, gentamycin, tetracycline, ciprofloxacin and amikacin and presence of multi-drug resistant strains were the most commonly detected characters in the STEC strains of hospital foods. Presence of O157 serogroups, EHEC strains, animal-based antibiotics and even antibiotic resistance genes which encode resistance against animal-based antibiotics in cooked foods showed insufficiency of cooking time and temperature in the kitchens of hospitals. It seems that there were no strict supervisions on the principles of food hygiene in Iranian hospitals. Due to the low levels of STEC resistance against imipenem, meropenem, streptomycin and cefotaxim antibiotics, occurrence of food poisonings due to the STEC strains in tested Iranian hospitals can be treated with their regular prescription. Attentions to the results of disk diffusion method and principles of hazard analysis and critical control point (HACCP) system can reduce the risk of STEC strains in hospital food stuffs.

## Abbreviations

AEEC: Attaching and effacing E. coli
E. coli: Escherichia coli
Eae: Intimin
EHEC: Enterohemolysin or Enterohaemorrhagic Escherichia coli
Ehly: Hemolysin
HC: Hemorrhagic Colitis
HUS: Hemolytic Uremic Syndrome
PCR: Polymerase Chain Reaction
SPSS: Statistical Package for the Social Sciences
STEC: Shiga toxin producing Escherichia coli
Stx: Shiga toxin
